# Practical Items

**Published:** 1852-01

**Authors:** 


### ARTICLE VIII.

### PRACTICAL ITEMS.

*Muriated Tincture of Iron in Erysipelas.* By Dr. Bell, [Edinburg Monthly Journal of Medical Science.]

Act freely on the bowels, and if the erysipelas is mild give 15 drops of the muriated tincture of iron in water every two hours until the disease is completely removed. When the attack threatens to be more severe, increase the dose to 25 drops every two hours, and persevered in night and day, however high the fever and delirium. The only local application necessary, are hair powder and cotton wadding. The bowels should be attended to throughout the disease. The tincture has been prescribed in idiopathic erysipelas, and in that resulting from external injury with the most satisfactory results; and it has been found equally efficacious at every period of life, from early infancy to advanced age. It not only removes erysipelas in a remarkably short time without weakening the patient, but it effects such an improvement in the system, that those who are subject to periodical attacks of the disease are rendered much less liable to have a return. It is a remarkable circumstance in the administration of this valuable remedy in the erysipelatous diathesis, that although given in much larger and more frequently repeated doses than have boen recommended in our dispensatories, it never produces headache, and when this symptom is present it quickly relieves it; at the same time, it reduces and regulates the pulse; thus showing that in this state of the system, it has a soothing and sedative, as well as an alterative effect.---*In N. Lancet.*

*Collodion in Ingrowing Toe-Nail.* By M. Meynier.—Press down the fleshy portion, and pour between it and the edge of the nail a small quantity of collodion; this soon solidifies induces rapid cicatrization of the ulceration, and when the disease does not arise from an abnormal shape of the nail, procures a cure.---*Ibid.*

*Pruritus of the Genital, Anal, and Axillary Regions.* By Dr. TOUNIE, [in L' Union Medicale.]

The following preparations are employed in the treatment of this troublesome affection : 1. An ointment of calomel, (4 to 6 parts calomel to 30 of axunge ; 2. A powder composed of ¾ of starch and ¼ of camphor, well pulverized and mixed. The proportion of calomel may be increased in the ointment, and that of camphor in the powder, according to the obstinacy of the disease. If the diseased parts are covered with scales or dry crusts, promote their separation by cata-plasms and emollient baths, then apply frictions twice a day with the ointment, and after the frictions, sprinkle the parts with the starch and camphor powder. Both remedies are to be employed ; the ointment alone is inefficacious, and the powder without the ointment, allays the itch but does not cure. These remedies exert no influence over the itching of the genitals that frequently accompanies the pregnant state.---*Ibid.*

*New Plan of Ligaturing Nævi Materni.* By Mr. ERICHSEN, [in London Loncet.]

Four cases are reported in which the writer resorted to a new plan for ligaturing nævi ; this mode is best adapted for elongated flat nævi, and in all those in which the ordinary ligatures cannot be applied without enclosing an undue quantity ot integument. The mode of operating is as follows ; make a puncture about an eight of an inch above the tumor, with a blunt-eyed probe, push the head across the base of the mass ; cut down upon its point when it projects below the tumor and then draw it across ; the transverse threads are to be carried through in a similar manner ; tie the knots in the usual way and you firmly and effectually strangulate the mass. The threads and sloughing mass will separate in a few days, leaving a healthy granulating surface, which speedily cicatrizes.---*Ibid.*

*Iodide of Potassium in Asthma.* (Dr. DEANE, in the Stethoscope.)—Three cases are related in which the administration of this remedy was followed with the most marked, and apparently permanent relief. The first is that of a clergyman, who had labored under the disease for several years ; by ta-ing the iodide he was immediately relieved, and was always able to ward off an attack by resorting to this remedy.

Case 2d. A young man aged 16 years, was affected with asthma for seven or eight years ; he took five grains of the iodide every two hours, and he was much relieved the next morning, the benefit being manifest after the exhibition of the second or third dose. Whenever threatened with a paroxysm, the immediate use of the potash prevented it.

Case 3d. A woman aged 32, suffered from regular attacks of asthma in the month of May, which had recurred for eight years, and was supposed to be occasioned by the odour of flowers. She took eight grains of the hydriodate of potash every four hours ; the symptoms were greatly mitigated during the next twenty-four hours, and after using the remedy in this way for three days, she was so much relieved that further treatment was discontinued.—*Ibid.*

*Modification of the Operation for Hare-Lip.* (Mr. Coste.) —To obviate the notch that remains after the ordinary plan of operating for this deformity, more especially in the simple kind, proceed as follows : cut a horizontal flap in the red part of the lip on one side, and a kind of half mortise on the other ; secure the flap and mortis by twisted suture, and by one of the diminutive sprig forceps called "serre fines ;" place a transverse needle a little higher up, and make no application whatever that the progress may be more accurately watched. —*Ibid.*

*Yeast Mixture in Petechial Typhus.* (Dr. Jones, in Dublin Quart. Jour. of Med.) Dr. J. speaks very highly of the stimulating and antiseptic properties of the following mixture in cases of typhus attended with petechiæ and other forms of passive hemorrhages :

℞. Cerevisiæ fermenti ℥x ;
Camphoræ ʒss ;
Ætheris nitrici ʒiv. ℥j to be taken every first, second or third hour. This removes the dark livid hue of the skin within a few hours ; administered in cases of dysentery attended with great fetor of the dejecta, it has speedily removed all odor, and at the same time rather counteracted the frequency of the discharges from the bowels.—*Ibid.*

*External Pressure in difficult Parturition.* (Boston Med. Jour.) It often happens that a woman in labor lack but very little of being able to evacuate the uterus by her own expulsive force, and the little force that she wants may be most conveniently and safely rendered by a judicious swathing of the abdomen. The end may be obtained in this way. Take for a swathe, a sheet, and fold it on one direction till it is reduced to about a quarter of a yard wide, retaining its whole length in the other direction. Lay this smoothly on the couch so that the woman can lay across it on her side. Then raise up the two ends of the swathe and bring them over her so as to cross on her hips, and give the ends to two assistants. If it is well adjusted they may use considerable force without any inconvenience to the patient, but rather the contrary. It is the most comfortable support to the back that she can have, and every pound of pressure smoothly and justly made on the abdomen is at least as good as so much traction on the child.

Another important consideration is that the expulsive action of the uterus is by this means increased, as is sometimes done by friction. The writer has resorted to this means in a large number of cases with decided advantage, and has not in a single instance met with any untoward circumstances, altho' the force applied has in some cases been about as much as two assistant women could apply. It may sometimes save the use of the forceps or of "turning," and is a less serious undertaking even with the smallest experience and judgment.—*Ibid.*

*Ergot in Visceral Engorgements.* (Dr. Barbieri, in Bull. delle Sc. Med. vol. xxvii.)—The writer has derived great benefit from the prolonged internal use of ergot of rye in hæmoptysis and incipient phthisis; and has obtained the same satisfactory results by its external application in splenic and hepatic engorgements, due for the most part to miasmatic causes. He either employs the pulverised ergot alone, ʒj to ℥j of simple ointment, or, when he wishes to produce more energetic cutaneous action, he adds five drops of croton oil, and in very serious cases five drops of creasote as well.—*Ibid.*

*Hemorrhoids treated by Nitric Acid.* (Dr. THWEATT, in the Stethoscope.) The patient had been affected for fifteen years with bleeding piles, and with a prolapsus recti on each attempt at stool. Having submitted to every plan of treatment without the slightest benefit, Dr. T. recommended cauterization with nitric acid. It was applied by penciling the tumors until it produced a change of color. The operation produced very little pain, and the parts were dressed with lint and sweet oil. On the second day he was entirely free from pain; the piles were less congested and slightly diminished in size; the bright red color was changed to a dirty brown. He was made to strain at stool, which act brought to view tumors situated higher in the rectum. These were cauterized as at first, a straining, burning pain excited. On the third day, the piles were again touched. A short time after the last penciling, he was cured; no tumors, either internal or external, and there was no further prolapse of the gut.—*Ibid.*

*Very minute doses of Tartar Emetic in Phthisis and Asthma.* (M. BERNARDEAU, in Bull. de Therap.) The writer some time since published the results of the general benefit he has obtained from the exhibition of very minute doses of tartar emetic in the hectic of phthisis. He has, since that period, used it in other stages of tuberculization, and in several cases of asthma with excellent effects. He gives from three to six pills in the twenty-four hours, each pill containing one twenty-fifth of a grain. The cough, dyspnœa, and inordinate action of the heart become calmned, and in fact, all the good effects of morphia, without its inconveniences seem to be produced. —*Ibid.*

*Pumpkin Seeds a Remedy for Tape-Worm.* (Dr. F. CRAGIN, in Boston Med. Jour.)—The patient took undried acorn or marrow squash seeds, followed in one hour and a half after with ℥vj of castor oil in two spoonfuls of Holland Gin. He drank very little water twice, drank and eat nothing else till noon, when occurred a liquid discharge containing the squirming worm about one-third of an inch broad at one end, and tapering down to nothing at the other.—*Ibid.*

